# Staff management in Paediatric Primary care setting

**DOI:** 10.1186/1824-7288-41-S2-A39

**Published:** 2015-09-30

**Authors:** Luigi Greco

**Affiliations:** 1 Family Paediatrician, ASL della Provincia di Bergamo, Bergamo, Italy

## 

In the last 6-7 years there has been a progressive increase in the use of nurses or secretaries in Paediatric Primary care setting.

Regional incentives (few) and the rising (remarkable) of the workload and patients’ requests, convinced about the 40% of the italian Family Paediatricians (FP) to hire new staff. In the 70% of cases the employers were organized in working group in one private practice that, thanks to the costs’ sharing, frequently had nurses in their staff (the data represent the situation at the end of 2013 and were obtained asking directly FP's professional organizations). Solo Paediatricians and Associated Paediatricians (that work in several private practices in coordination each other), on the other hand, hired more frequently secretaries with a working time generally reduced if compared to working groups’ colleagues.

Economic factors (the fear to be unable to fulfill the expenses related to hiring) and those concerning organization and management (the not perfect knowledge of how best to use the staff) are the main causes that prevent most of Paediatricians to employ [1].

Who decided to use co-workers, did it to delegate certain duties, to filter patients’ requirements, to share work experience [1].

Secretaries can officiated the following tasks: sorting incoming calls, appointments, advise on admission to medical consultation, set up patient's recording (1st consultation), illustration and delivery of the services charter, delivery questionnaires, registration of diagnostic services, recurrent prescriptions, preparation of medical certificates, patients’ active call for programmed medical examinations, billing, payment deadlines and commitments, check of the monthly payment slips obtained by the Health Service, registration deadlines of drugs and medical devices, suppliers list, mantainance men and contracts list, health servicices summaries, statistics, chart statistic diagrams, waiting room monitoring (games, cartoons, magazines, books, etc.), appointment calendar update.

Nurses can perform the following functions: Telephone Triage; Telephone advices based on defined protocols that are recorded in the electronic patient health card, auxological recordings, performing diagnostic tests and screening (urine examination, rapid Strep test, RCP, Blood Count, Prick Test, oximetry, spirometry, eye test, Lang test, Boel test, Plicometry, etc.), infant care, clinical and epidemiological research, health education messages in the waiting room, drugs and medical devices deadlines monitoring, provisions supervision, medical equipment efficiency preservation, health care protocols update and recording, check office's e-mails.

In the near future the decrease of FP's number, the need to take care of a larger number of patients and to organize work in some way of collaboration, will push more and more colleagues to hire staff.
